# Prognostic Implication and Survival Outcomes of Perioperative Blood Transfusion on Urological Malignancies Undergoing Radical Surgical Intervention

**DOI:** 10.30699/ijp.2023.553040.2887

**Published:** 2023-03-23

**Authors:** Sujata Mallick, Mahasweta Mallik, Puskar Shyam Chowdhury

**Affiliations:** 1 *Department of Pathology, KPC Medical College, * *West Bengal University of Health * *Science, Kolkata, India*; 2 *Department of Pathology, NSMCH, Bihta, Aryabhatta University, Patna, India*; 3 *Department of Urology, KPC Medical College, Professor, West Bengal University of Health Science, Kolkata, India*

**Keywords:** Cystectomy, Nephrectomy, Perioperative blood transfusion, Prostatectomy, Urological malignancies

## Abstract

**Background & Objective:**

Background and objective: Perioperative blood transfusion (PBT) during radical urological surgeries has been associated with an increased incidence of complications. The present study analyzes the outcome of perioperative blood transfusion (PBT) and the prognostic implications after radical surgeries on patients with malignant urological tumors.

**Methods:**

Our retrospective study included 792 cases of partial or radical nephrectomy /cystectomy/prostatectomy surgeries for kidney/bladder/ prostate carcinoma from 2012 to 2022. Data on preoperative, intraoperative, and pathological parameters were evaluated. PBT was taken as a period of transfusion of allogenic RBC during/preoperative/postoperative surgeries. The effect of PBT on oncological parameters like recurrence-free survival (RFS), overall survival (OS), and cancer-free survival (CSS) was compared using univariate cox regression analysis (Odds ratio, Hazard ratio).

**Results:**

PBT was applied on 124 (20.6%) patients of nephrectomy, 54 (46.5%) patients of cystectomy, and 23 (31%) patients of prostatectomy. The baseline characteristics of the cohort study found symptomatic patients with older age and other co-morbidities to be transfusion-dependent. Also, the patients undergoing radical operations with more blood loss and advanced tumor stage were more likely to receive PBT. PBT was significantly associated with survival outcomes (*P*<0.05) in nephrectomy and cystectomy cases but independent of association in prostatectomy cases.

**Conclusion:**

The result of this study concludes that in nephrectomy and cystectomy operations, PBT had a significant association with cancer recurrence and mortality; however, in prostatectomy cases, no significant correlation was noted. Thus, proper criteria to prevent the unnecessary use of PBT and more defined parameters for transfusion are needed to improve postoperative survival. Autologous transfusion should be considered more frequently. However, more extensive studies and randomized trials are needed in this area.

## Introduction

It is a known fact that transfusion of red blood cells suppresses the immune system, leading to increased tumor recurrence in patients with carcinoma undergoing surgery. Improved surgical approaches have recently led to less blood loss, especially in radical operations. Despite this, a substantial number of patients still need perioperative blood transfusions (1). These oncology patients risk hemorrhage and coagulation pitfalls due to tumor pathology and immune function impairment. Coagulopathy may be drug-induced (anticoagulants, anesthetic agents) and decreased host immunity caused by tissue injury. Preoperative treatment protocols (immune therapies, chemotherapy, radiotherapy), tumor proximity to vessels, difficulties in operative techniques, and intraoperative factors (temperature stabilization, hemodilution, metabolic disorders) may all influence blood loss during oncological surgery (2). Urological cancers is an umbrella term for all urological malignancies comprising predominantly carcinoma of the kidney, urinary bladder, and prostate. These tumors require resection and perioperative blood transfusion (PBT) requirement is a predominant feature in these surgeries. Our study analyses the outcome of perioperative blood transfusion on tumor recurrence and prognosis in renal, prostate, and urinary bladder tumor resections.

## Material and Methods

Our study was done at KPC Medical College and Fortis Hospital, Kolkata, from 2012 to 2022. It was a descriptive retrospective study. We reviewed the nephrectomy, cystectomy, and prostatectomy patient database in the hospital's record section. Exclusion criteria included insufficient data on patient demographics and patients with incomplete information about PBT. Diseases other than primary RCC, TCC, or prostatic carcinoma were also the exclusion criteria. Autologous blood transfusion was excluded due to very few cases. A total of 792 patients with urological malignancies were finally included in our study.

Financial and material support- Since it was a retrospective study based on the patient database, there was no requisition for a financial grant.

**Research Ethics and Patient's Consent**

Our study followed the standards according to the Universal ethical norms. The routine patient's consent before surgical procedure and blood transfusion was mandatory. We were exempted from getting informed consent as our institutional scientific committee permitted the use of patient's information retrieved from the hospital's database. Personal identification of the patients was deleted. The data collected was evaluated and recorded by expert genitourinary pathologists with double blinding according to the institution's standard operating procedure (SOP).

The PBT (Perioperative blood transfusion) group was taken as patients who received allogenic blood transfusion comprising packed RBCs or whole blood within 7 days before/during surgery or within the postoperative hospitalization time. Transfusion of fresh frozen plasma or platelets was not included due to fewer cases. Non-PBT group was patients who did not receive blood transfusion for the required surgery. The decisions for administering blood depended on the clinical evaluation of the anesthesiologist and surgeons.

Some studies show that the administration of older stored blood units causes cellular and humoral changes. Recently acquired blood does not affect the function of the exposed lymphocytes. However, we could not categorize the blood as 'older,' 'middle,' and younger blood due to insufficient data.

All the surgical interventions and blood transfusions were in the pre-COVID era. However, the follow-up of the patients received a setback due to SARS-COV 2 pandemic. The online consultation mode was utilized for patients who could not attend the department physically. The proper COVID protocol norms were implemented for physical checkups as laid down by the institution. 

Follow-up was done quarterly during the first five years after the oncosurgery and then yearly after that for the next 5 years. The patients who came for postoperative follow-up for less than 6 months were excluded from our study.

Considering the cases lost to follow-up, we included only patients who had either tumor recurrence or followed up for more than six months.

The follow-up was done with 

a) routine blood tests 

b) radiological diagnosis like computed tomography (CT)/ magnetic resonance imaging (MRI) as per requirement.

Clinical data were reviewed and collected, including:

1) Age, gender, body mass index (BMI), diabetes, hypertension, or presence of end-stage renal disease (ESRD), Eastern Cooperative Oncology Group (ECOG) performance and symptoms at presentation 

2) Biochemical parameters like hemoglobin level and creatinine 

3) Surgical data like method and time taken by the operative procedure, amount of blood loss, and perioperative blood transfusion (PBT) 

4)Pathological factors like the presence of Tumor, Necrosis, Metastasis (TNM) stage, histopathology of the tumor, nuclear grading, size of the tumor mass, and other histological parameters 

5) Follow-up data like 1. disease relapse, 2. death due to the disease

TNM staging was done using American Joint Committee on Cancer (AJCC 8^th^ edition) guideline.

The outcomes were analyzed based on the endpoints, that is, overall survival (OS), cancer-specific survival (CSS), and recurrence-free survival (RFS) at 3, 5, and 10 years. "Recurrence Free Survival was defined as the period from surgery to the first presentation of disease recurrence. Cancer-Specific Survival and Overall Survival were taken to be the time from surgery to tumor-related death or any cause death, respectively" (1). Whether they had a tumor or not, patients who did not die were censored from the survival analysis. The data pertaining to the cause of death was retrieved from the medical documentation. 

**Statistics**

The parameters included in our study were predominantly a comparison between PBT and non-PBT patients. The methods used were Chi-square exact test for categorical variables, which were presented as absolute numbers and relative percentages. The Mann-Whitney test was applied and denoted as the interquartile range (IQR) for continuous variables. The preoperative conditions were analyzed using univariate logistic regression. We took the help of a log-rank test to find the X^2^ value. The P-value and the confidence intervals were also calculated using the same method. Odds ratio estimation (OR), its standard error, and 95% confidence error were assessed using the Cox proportional hazard models according to Altman, 1991. All statistics were calculated using DBM SPSS Version 21.0 (SPSS Inc., Chicago, Ill., USA) and MedCalc software. The P-value was calculated regarding Sheskin (2004). Accordingly, in this method, a normal standard deviation (z-value) was calculated, as In(OR)/SE{In(OR)}; the P-value was considered as an area of the normal distribution that falls outside ±z. The P-value of ˂0.05 was taken to be significant**.**

## Results

**Fig. 1:**
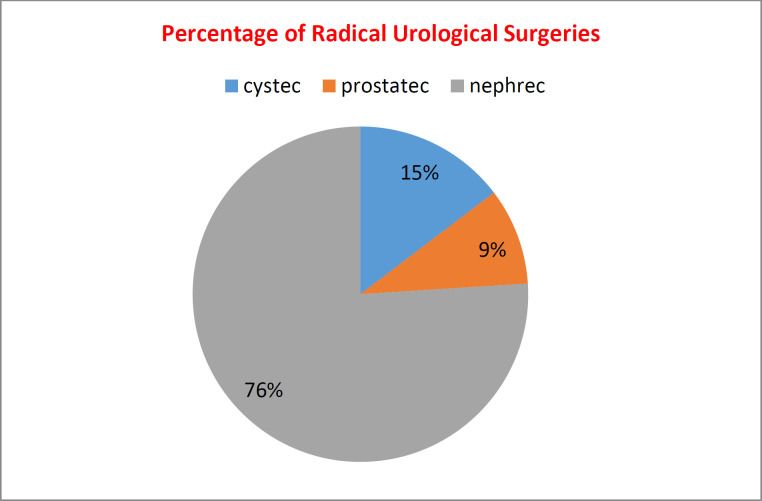
Distribution of cases according to Radical surgeries; cystec-cystectomy, prostatec-prostatectomy, nephrec-nephrectomy

Among 792 patients with urological malignancies, 602 (76%) underwent radical nephrectomy, 116 (15%) underwent radical cystectomy, and 74 (9%) underwent radical prostatectomy (Figure 1).

**Fig. 2:**
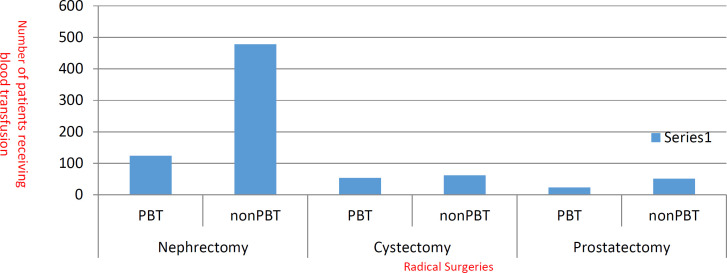
Comparison of study cohort among those receiving PBT and those not receiving PBT; PBT-Perioperative Blood Transfusion

Among all 602 patients undergoing nephrectomy, 124 received PBT (20.6%) with a median unit of 1-2 whole blood or packed RBC units (pRBCs) (IQR-1-3). Likewise, among 116 cystectomy patients, 54(46.5%) received PBT with 2-3 whole blood or pRBC units (IQR-1-4). Among 74 patients undergoing prostatectomy, PBT was performed in 23(31%) patients with whole blood or pRBC transfusion on an average of 1-2 units (IQR-1-3) (Figure 2). The need for transfusion occurred in the preoperative, intraoperative, or postoperative period. In nephrectomy patients, blood transfusion was primarily required intraoperatively. In cystectomy patients, blood transfusion was required majorly in pre and postoperative periods. In radical prostatectomy, the requirement was relatively less and mostly postoperatively (Table 1).

The parameters of the study group and the comparison between the PBT and non-PBT groups are listed in Tables 2, 3, and 4.

**Table 1:** Study cohort receiving the average blood volume, type of units transfused, and interval of transfusion; pRBC- packed red blood cells

Type of surgery	Type of units transfused	Time Interval of Transfusion		
		Preoperative	Intraoperative	Postoperative
Nephrectomy- 124cases Average (1-2) units Transfused	Whole blood 37	9	21	**13**
	pRBC 87	11	25	**17**
Cystectomy- 54 cases Average(2-3)units Transfused	Whole Blood 16	5	12	**10**
	pRBC 38	6	13	**19**
Prostatectomy- 23cases Average(1-2)units Transfused	Whole Blood 7	1	2	**4**
	pRBC 16	1	3	**12**

**Table 2:** Parameters of the study group and comparison according to the receipt of PBT for nephrectomy surgeries; PBT (Perioperative Blood Transfusion), ESRD (End Stage Renal Disease), ECOG (Eastern Cooperative Oncology Group), ccRCC (Clear cell Renal Cell Carcinoma), CSS (Cancer-specific Survival)

Variable NEPHRECTOMY 602	Total 602	Non-PBT-478(79.4%)	PBT-124(20.6%)
Preoperative clinical parameters			
Symptomatic presentation			
Asymptomatic	418 (69.4%)	359 (75.1%)	**59 (47.6%)**
Symptomatic	184 (30.5%)	119 (24.9%)	**65 (52.4%)**
Age at surgery	61 (55-72%)	64 (58-72%)	**59 (55-71)**
Sex			
Male	481 (79.9%)	386 (80.7%)	**95 (76.6%)**
Female	121 (20.1%)	92 (18.8%)	**29 (23.4%)**
Body mass index(BMI)	23.7 (21.1-27.4)	24.5 (21.8-27.4)	**23.2 (21.1-25.8)**
Preoperative hemoglobin	12.1 (10.8-15.2)	13.6 (11.3-15.2)	**11.4 (10.8-13.2)**
Preoperative serum creatinine	1.1 (0.6-1.6)	0.8 (.6-1.3)	**1.2 (0.7-1.6)**
Smoking status			
No	377 (62.6%)	288 (60.3%)	**89 (71.8%)**
Yes	225 (37.3%)	190 (39.7%)	**35 (28.2%)**
ECOG performance status			
0	437 (72.6%)	361 (82.6%)	**76 (61.3%)**
1	123 (20.4%)	91(19.0%)	**32 (25.8%)**
2	23 (3.8%)	14 (2.9%)	**9 (7.3%)**
3	09 (1.5%)	6 (1.3%)	**3 (2.4%)**
Missing/unknown	10 (1.7%)	6 (1.3%)	**4 (3.2%)**
Hypertension			
No	245 (40.7%)	177 (37.0%)	**68 (54.8%)**
Yes	357 (59.3%)	301 (63%)	**56 (45.2%)**
Diabetes mellitus			
No	446 (74.0%)	355 (74.3%)	**91 (73.4%)**
Yes	156 (25.9%)	123 (25.7%)	**33 (26.6%)**
ESRD			
No	577 (95.8%)	464 (97%)	**113 (91.1%)**
Yes	25 (4.25%)	14 (2.9%)	**11 (8.9%)**
Intraoperative parameters			
Surgical approach 1			
Laparoscopic	476 (79.0%)	385 (80.5%)	**91 (73.4%)**
Open	126 (20.9%)	93 (19.5%)	**33 (26.6%)**
Surgical approach 2			
Radical	373 (62%)	296 (61.9%)	**77 (62.1%)**
Partial	221 (36.7%)	180 (37.7%)	**41 (33.1%)**
Partial to Radical	08 (1.3%)	2 (0.4%)	**6 (4.8%)**
Operation time (minutes)	150 (110-240)	160 (115-170)	**200 (110-280)**
Estimated blood loss (mL)	300 (100-1200)	200 (100-300)	**600 (300-1200)**
Pathological parameters			
Histological subtype			
cc RCC	478 (79.4%)	382 (79.9%)	**96 (77.4%)**
Non-cc RCC	124 (20.6%)	96 (20.1%)	**28 (22.6%)**
Pathologic tumor(pT) stage			
pT1	450 (74.75%)	387(80.9%)	**63(50.8%)**
pT2	57 (9.5%)	38 (8%)	**19 (15.3%)**
pT3	89 (14.8%)	48 (10%)	**41 (33%)**
pT4	06 (1.0%)	5 (1%)	**01 (0.8%)**
Fuhrmann nuclear grade			
Grade1	38 (6.31%)	33 (6.9%)	**5 (0.1%)**
Grade2	303 (50.3%)	257 (53.8%)	**46 (37%)**
Grade3	217 (36.0%)	162 (34%)	**55 (44.4%)**
Grade4	39 (6.5%)	21 (4.4%)	**18 (14.5%)**
Missing/unknown	5 (0.8%)	5 (1%)	**0 (0%)**
Tumor size(cm)	4.3 (2-15)	3.5 (2-7)	**5.6(3-15)**
Pseudosarcomatous component			
Absent	591 (98.2%)	471 (98.5%)	**120 (96.8%)**
Present	11 (1.8%)	07 (1.5%)	**49 (39.5%)**
Tumor necrosis			
Absent	413 (68.6%)	349 (73%)	**64 (51.6%)**
Present	189 (31.4%)	129 (27%)	**60 (48.4%)**
Pathologic nodal (pN) stage			
pN0	76 (12.6%)	48 (10%)	**28 (22.6%)**
pN1	12 (2.0%)	05 (1%)	**07 (5.6%)**
pNx	514 (85.4%	425 (88.9%)	**89 (71.8%)**
Postoperative follow-up parameters			
Time to recurrence(months)	30 (20-45)	42 (30-45)	**24 (20-33)**
Recurrence result			
No recurrence	555 (92.2%)	458 (95.8%)	**97 (78.2%)**
Recurrence	47 (7.8%)	20 (4.2%)	**27 (21.8%)**
Median follow-up duration(months)	48 (16-116)	54 (25-115)	**42 (16-116)**
Overall survival result			
Alive	515 (85.6%)	426 (89.1%)	**89 (71.8%)**
All-cause death	87 (14.5%	52 (10.9%)	**35 (28.2%)**
CSS result			
Alive or other cause of death	552 (91.7%)	454 (95%)	**98 (79%)**
Cancer-specific death	50 (8.3%)	24 (5%)	**26 (21%)**

**Table 3:** Parameters of the study group and comparison according to the receipt of PBT for cystectomy surgeries; PBT (Perioperative Blood Transfusion), ECOG (Eastern Cooperative Oncology Group), TCC (Transitional Cell Carcinoma), CSS (Cancer-specific Survival)

Variable CYSTECTOMY 116	Total 116	Non-PBT-62(53.4%)	PBT-54(46.5%)
Preoperative clinical parameters			
Symptomatic presentation			
Asymptomatic	60 (51.7%)	43 (69.3%)	17 (31.5%)
Symptomatic	56 (48.3%)	19 (30.6%)	37 (68.5%)
Age at surgery	55 (45-80)	60 (47-80)	53 (45-72)
Sex			
Male	84 (72.4%)	46 (74.2%)	38 (70.4%)
Female	32 (27.6%)	16 (28.5%)	16 (29.6%)
Body mass index(BMI)	22.8 (21.4-27)	23 (21.6-27)	22 (21.4-25.6)
Preoperative hemoglobin	11 (9.8-15.2)	13(11-15.2)	10.5(9.8-13.5)
Preoperative serum creatinine	1 (0.83-1.3)	1 (0.83-1.2)	1.02 (0.85-1.3)
Smoking status			
No	73 (63.0%)	40 (64.5%)	33 (61.1%)
Yes	43 (37.0%)	22 (35.5%)	21 (38.9%)
ECOG performance status			
0	79 (68.1%)	46 (74.2%)	33 (61.1%)
1	25 (21.6%)	12 (19.4%)	13 (24%)
2	05 (4.3%)	1 (1.6%)	4 (7.4%)
3	02 (1.8%)	1 (1.6%)	1 (1.9%)
Missing/unknown	05 (4.3%)	2 (3.2%)	3 (5.6%)
Hypertension			
No	63 (54.3%)	37 (59.7%)	26 (48.2%)
Yes	53 (45.7%)	25 (40.3%)	28 (51,9%)
Diabetes mellitus			
No	92 (79.3%)	51 (82.3%)	41 (75.9%)
Yes	24 (20.7%)	11 (17.7%)	13 (24.1%)
Intraoperative parameters			
Surgical approach 1			
Laparoscopic	26 (22.4%)	18 (29%)	8 (14.8%)
Open	90 (77.6%)	44(71%)	46 (85.2%)
Operation time(minutes)	310 (220-480)	280(220-360)	32 0(240-480)
Estimated blood loss(mL)	500 (150-1200)	300 (150-400)	900 (400-1200)
Pathological parameters			
Histological subtype			
TCC	113 (97.4%)	61 (98.9%)	52 (96.3%)
Others			
Adenocarcinoma	01 (.9%)	1 (1.1%)	0
Squamous cell carcinoma	02 (1.7%)	0	2 (3.7%)
Pathological Tumor (pT) stage			
pT1	74 (63.8%)	48 (77.4%)	26 (48.1%)
pT2	28 (24.1%)	11 (17.7%)	17 (31.5%)
pT3	11 (9.5%)	02 (3.2%)	09 (16.7%)
pT4	03 (2.6%)	01 (1.6%)	02 (3.7%)
Tumor grade			
Non-invasive			
Low grade High grade	26 (22.4%)	05 (8.1%)	21 (38.9%)
	37 (31.9%)	26 (41.9%)	11 (20.4%)
Invasive			
Low grade Without deep muscle involvement With deep muscle involvement			
	08 (6.9%)	07 (11.3%)	01 (1.9%)
	18 (15.5%)	13 (20.9%)	05 (9.3%)
High grade Without deep muscle involvement With deep muscle involvement			
	11 (9.5%)	08 (12.9%)	03 (5.6%)
	14 (12.0%)	02 (3.2%)	12 (22.2%)
Missing	02 (1.7%)	01 (1.6%)	01 (1.9%)
Tumor size(cm)	2.5 (1-4.6%)	1.4 (1-1.9)	4.2 (3.8-4.6)
Pathologic nodal (pN) stage			
pN0	95 (84.8%)	59 (95.2%)	36 (66.7%)
pN1	15 (12.9%)	02 (3.2%)	13 (24%)
pNx	06 (5.2%)	01 (1.6%)	05 (9.3%)
Postoperative follow-up parameters			
Time to recurrence(months)	20 (14-29)	24 (16-29)	18 (14-22)
Recurrence result			
No recurrence	78 (67.2%)	40 (64.5%)	38 (70.4%)
Recurrence	28 (24.1%)	12 (19.4%)	16 (29.6%)
Median follow-up duration(months)	76 (18-115)	74 (28-112)	78 (18-115)
Overall survival result			
Alive	84 (72.4%)	44 (70.9%)	40 (70%)
All-cause death	22 (19.0%)	08 (12.9%)	14 (25.9%)
CSS result			
Alive or other cause of death	90 (77.6%)	46 (74.2%)	44 (81.5%)
Cancer-specific death	16 (13.8%)	06 (9.7%)	10 (18.5%)

**Table 4:** Parameters of the study group and comparison according to the receipt of PBT for prostatectomy surgeries; PBT (Perioperative Blood Transfusion), ECOG (Eastern Cooperative Oncology Group), CSS (Cancer-specific Survival)

Variable PROSTATECTOMY 74	Total 74	Non-PBT-51(68.9%)	PBT-23(31%)
Preoperative clinical parameters			
Symptomatic presentation			
Asymptomatic	66 (89.2%)	46 (90.2%)	20 (87%)
Symptomatic	08 (10.8%)	05 (9.8%)	03 (13%)
Age at surgery	60.2 (55-79)	60 (58-78)	61 (55-79)
Body mass index(BMI)	27 (25.2-28.3)	27 (25.5-28.2)	26 (25.2-28.3)
Preoperative hemoglobin	10.0 (7-13)	10.5 (7-12)	9 (7.5-13)
Preoperative serum creatinine	1.0 (0.6-2.8)	1.0 (0.6-1.8)	1.1 (1-2.8)
Smoking status		
No	51 (68.9%)	23 (45%)	18 (78.3%)
Yes	23 (31.1%)	11 (21.6%)	05 (21.8%)
ECOG performance status		
0	54 (72.9%)	38 (74.5%)	16 (69.6%)
1	12 (16.2%)	8 (15.7%)	4 (17.4%)
2	03 (4.1%)	2 (3.9%)	1 (4.4%)
3	0 (0)	0	0
Missing/unknown	05 (6.8%)	3 (5.9%)	2 (8.7%)
Hypertension			
No	40 (54.0%)	27 (52.9%)	13 (56.5%)
Yes	34 (45.9%)	24 (47.1%)	10 (43.5%)
Diabetes mellitus			
No	47 (63.5%)	31 (60.8%)	16 (69.6%)
Yes	27 (36.5%)	20 (39.2%)	07 (30.4%)
Intraoperative parameters			
Surgical approach			
Laparoscopic	46 (62.2%)	35 (68.6%0	11 (47.8%)
Open	28 (37.8%)	16 (31.4%)	12 (52.2%)
Operation time(minutes)	200 (130-360)	210 (150-360)	178 (130-310)
Estimated blood loss(mi)	550 (100-1400)	600 (200-1400)	400 (100-900)
Pathological parameters			
Histological subtype			
Adenocarcinoma	68 (91.9%)	47 (92.2%)	21 (91.3%)
Others	06 (8.1%)	04 (7.8%)	02 (8.7%)
Pathologic tumor (pT) stage			
pT1	15 (20.3%)	10 (19.6%)	05 (21.7%)
pT2	51 (68.9%)	35 (68.6%)	16 (69.6%)
pT3	08 (10.8%)	06 (11.8%)	02 (8.7%)
pT4	0 (0)	0	0
Gleason score			
Score 6	21 (28.4%)	14 (27.5%)	07 (30.4%)
Score 7	26 (35.1%)	18 (35.3%)	08 (34.8%)
Score 8	15 (20.3%)	10 (19.6%)	05 (21.7%)
Score 9	10 (13.5%)	07 (13.7%)	03 (13%)
Score 10	02 (2.7%)	01 (2%)	01 (4.4%)
Missing/unknown	02 (2.7%)	01 (2%)	01 (4.4%))
Pathologic nodal (pN) stage			
pN0	56 (75.7%)	38 (74.5%)	18 (78.3%)
pN1	10 (13.5%)	07 (13.7%)	03 (13%)
pNx	08 (10.8%)	06 (11.8%)	02 (8.7%)
Postoperative follow-up parameters			
Time to recurrence(months)	30 (9-58)	26 (10-58)	34 (9-54)
Recurrence result			
No recurrence	59 (79.7%)	40 (78.4%)	19 (82.6%)
Recurrence	15 (20.3%)	11 (21.6%)	04 (17.4%)
Median follow-up duration(months)	55 (26-116)	56 (26-116)	54 (34-112)
Overall survival result			
Alive	59 (79.7%)	40 (78.4%)	19 (82.6%)
All-cause death	15 (20.3%)	11 (21.6%)	4 (17.4%)
CSS result			
Alive or other cause of death	65 (87.8%)	44 (86.3%)	21 (91.3%)
Cancer-specific death	09 (12.2%)	07 (13.7%)	02 (8.7%)

Baseline characteristics of the study cohort showed that those receiving PBT were more likely to be symptomatic (52.4%, 68.5%, and 13%), respectively, for nephrectomy, cystectomy, and prostatectomy. Also, the patients in the older age group, those with lower BMI, lower hemoglobin value, and higher creatinine value, had more need for blood transfusion during the operation. Furthermore, the patients with a history of smoking, those with poor ECOG performance levels, and with co-morbidities like hypertension, diabetes mellitus, or ESRD were predisposed to be transfusion-dependent. Blood transfusion was a requisite in open surgeries, surgeries with a radical approach, surgeries needing longer operating time, and those with more blood loss. Patients presenting with worse tumor stage, greater nuclear grade, bigger tumor size, associated pseudosarcomatous areas, necrosis (for nephrectomy specimen), lymph node invasion, and higher Gleason score (for prostatectomy specimen) were more prone to receive a perioperative blood transfusion.

The median follow-up period between both groups (42 months vs. 54 months for nephrectomy, 78 months vs. 74 months for cystectomy, and 54 months vs. 56 months for prostatectomy) showed variation, which was statistically significant.

Postoperative recurrence in nephrectomy patients was recorded in 27 patients (21.8%). The median time was 24(IQR20-33 months) in the PBT group. Among the non-PBT group in nephrectomy patients, postoperative recurrence was seen in 20 patients (4.2%). The median time was 42(IQR30-45 months). In cystectomy patients, postoperative recurrence in the PBT group was 16 (29.6%). The median time was 18(IQR14-22 months). In the non-PBT group, recurrence was 12 (19.4%), and the median time was 24(IQR16-29 months). For prostatectomy patients, postoperative recurrence in the PBT and non-PBT groups was 4 (17.4%) and 11 (21.6%). In this group, the median time was 34(IQR9-54 months) and 26(IQR10-58 months), respectively.

**Table 5:** Univariate logistic regression analysis (Unadjusted Odds ratio) to assess risk factors for PBT; OR (Odds ratio), ECOG (Eastern cooperative oncology group), ESRD (End stage Renal disease), Hb (Haemoglobin)

Univariate logistic regression analysis or unadjusted Odds ratio demonstrated that symptomatic presentation, worst ECOG, hypertension, ESRD, higher creatinine value, and lower preoperative hemoglobin value (*P*<0.05) were independent preoperative etiological factors for PBT in nephrectomy cases. For cystectomy patients, symptomatic presentation and higher age (*P*<0.05) and for prostatectomy patients, only low preoperative Hb (*P*<0.05) were significant preoperative risk factors. Low body mass index for nephrectomy patients and symptomatic presentation for prostatectomy patients have equivocal significance (*P*±0.05) as a risk factor for PBT (Table 5).

**Nephrectomy**

**Table 6:** Cox proportional hazards regression model for survival outcomes (RFS, OS, CSS) in PBT receiving cohort for radical nephrectomy; RFS (Recurrence-free survival), OS (Overall Survival), CS (Cancer-specific survival), PBT (Perioperative Blood Transfusion), ECOG (Eastern Cooperative Oncology Group), BMI (Body Mass Index), N1 (Nodal metastasis)

Variable	RFS		OS		CSS	
	HR(95%CI)	P-value	HR(95%CI)	P value	HR(95%CI)	P-value
Symptomatic	2.402(1.616-3.574)	0.000	2.127(1.405-3.222)	0.0001	2.526(1.705-3.745)	0.00
Age	1.248(0.940-1.658)	0.100	1.259(0.939-1.689)	0.097	1.019(0.764-1.361)	0.894
BMI	1.180(0.823-1.693)	0.341	1.284(0.881-1.874)	0.158	1.201(0.839-1.720)	0.289
Hemoglobin	1.33(0.862-2.056)	0.158	1.318(0.829-2.047)	0.201	0.303(0.239-0.384)	0.00
ECOG						
1	1.081(0.625-1.702)	0.903	1.038(0.620-1.739)	0.885	1.143(0.687-1.906)	0.590
2	3.004(0.894-10.141)	0.016	3.191(0.837-12.168)	0.019	4.632(1.285-16.7)	0.0007
3	2.360(0.287-19.413)	0.306	3.589(0.506-25.469)	0.073	1.853(0.267-12.877)	0.453
Hypertension	0.805(0.610-1.065)	0.157	0.809(0.607-1.079)	0.178	0.668(0.503-0.889)	0.015
Diabetes mellitus	1.148(0.740-1.782)	0.518	1.058(0.668-1.677)	0.804	1.105(0.711-1.718)	0.647
Tumor diameter	0.674(0.496-0.918)	0.028	0.684(0.492-0.950)	0.046	0.620(0.454-0.847)	0.010
Pathological Tumor stage(pT)						
pT2	2.279(1.038-5.008)	0.0092	1.950(0.841-4.52)	0.057	1.853(0.779-4.410)	0.093
pT3	3.685(2.014-6.742)	0.00	4.239(2.240-8.025)	0.00	4.038(2.216-7.361)	0.00
pT4	0	0.357	1.595(0.119-21.309)	0.683	0	0.298
Nuclear grade						
Grade 3	1.195(0.825-1.734)	0.318	1.222(0.824-1.816)	0.287	1.281(0.874-1.878)	0.169
Grade 4	4.092(1.543-10.852)	0.00005	4.786(1.585-14.455)	0.00005	2.725(1.016-7.313)	0.008
Pseudo sarcoma	3.147(0.313-31.649)	0.184	2.393(0.120-47.731)	0.462	2.316(0.120-44.76)	0.480
Tumor necrosis	2.209(1.469-3.322)	0.0002	2.233(1.471-3.392)	0.00002	2.170(1.452-3.243)	0.00002
Stage N1	5.902(1.057-32.970)	0.002	6.382(0.9-45.277)	0.005	6.948(1.372-35.184)	0.0004

**Cystectomy**

**Table 7:** Cox proportional regression hazard model for survival outcomes (RFS, OS, CSS) in PBT cohort for radical cystectomy RFS (Recurrence-free survival), OS (Overall Survival), CS (Cancer-specific survival), PBT (Perioperative Blood Transfusion), ECOG (Eastern Cooperative Oncology Group), BMI (Body Mass Index), N1 (Nodal metastasis)

Variable		RFS		OS		CSS	
		HR (95%CI)	P-value	HR (95%CI)	P-value	HR (95%CI)	P-value
Symptomatic		2.180(1.199-3.965)	0.014	2.053(1.129-3.736)	0.021	2.090(1.187-3.682)	0.013
Age		1.203(0.671-2.158)	0.535	1.386(0.805-2.390)	0.239	1.425(0.828-2.456)	0.204
BMI		0.80(0.443-1.445)	0.462	0.825(0.471-1.445)	0.504	0;793(0.458-1.373)	0.411
Hemoglobin		0.701(0.391-1.259)	0.241	0.733(0.421-1.277)	0.280	0.708(0.411-1.220)	0.219
ECOG							
1	1.169(0.476-2.876)		0.732	1.21(0.514-2.849)	0.662	1.045(0.415-2.634)	0.924
2	inf		0.146	3.30(0.464-23.479)	0.272	inf	0.148
3	0		0.329	inf	0.294	Nan	
Hypertension	1.238(0.650-2.360)		0.516	1.265(0.695-2.301)	0.440	1.045(0.571-1.915)	0.885
Diabetes mellitus	1.315(0.522-3.316)		0.561	1.21(0.514=2.849)	0.662	1.161(0.473-2.856)	0.744
Tumor diameter	1.015(0.598-1.722)		0.955	0.993(0.596=1.656)	0.980	0.980(0.596-1.613)	0.936
Pathological Tumor stage							
pT2	1.637(0.723-3.709)		0.243	1.43(0.631-3.241)	0.392	1.140(0.504-2.583)	0.752
pT3	7.368(1.842-29.476)		0.028	8.80(2.379=32.553)	0.013	Inf	0.002
pT4	inf		0.146	1.1(0.069-17.642)	0.946	Inf	0.306
Tumor grade							
Low grade	0.280(0.114-0.690)		0.015	0.343(0.146-0.809)	0.028	0.232(0.101-0,536)	0.003
High grade	2.105(0.835-5.305)		0.127	1.65(0.686-3.968)	0.267	1.698(0.722-3.997)	0.232
Stage N1	10.526(3.227-34.335)		0.005	6.05(2.037-17.966	0.007	11.50(3.708=35.667)	0.003

**PROSTATECTOMY**

**Table 8.** Cox proportional regression hazard model for survival outcomes(RFS, OS, CSS) in PBT cohort for radical prostatectomy; RFS (Recurrence-free survival), OS (Overall Survival), CS (Cancer-specific survival), PBT (Perioperative Blood Transfusion), ECOG (Eastern Cooperative Oncology Group), BMI (Body Mass Index), N1 (Nodal metastasis)

Variable	RFS		OS		CSS	
	HR (95%CI)	P-value	HR (95%CI)	P-value	HR (95%CI)	P-value
Symptomatic	1.403(0.215=9.16)	0.709	1.578(0.323-7.707)	0.546	1.047(0.189-5.798)	0.957
Age	1.228(0.622-2.42)	0.540	1.263(0.651-2.452)	0.473	1.197(0.636-2.252)	0.565
BMI	0.842(0.381-1.861)	0.680	0.701(0.318-1.55)	0.414	0.785(0.379-1.630)	0.536
Hemoglobin	0.467(0.225-0.971)	0.084	0.323(0.151-0.697)	0.026	0.488(0.245-0.974)	0.082
ECOG						
1	1.053(0.260-4.261)	0.942	1.578(0.328-7.707)	0.546	1.676(0.415-6.777)	0.436
2	2.105(0.108-40.874)	0.590	0	0.329	1.047(0.093-11.778)	0.969
Hypertension	0.886(0.395-1.987)	0.774	1.114(0.490-2.537)	0.792	0.942(0.433-2.053)	0.883
Diabetes mellitus	0.561(0.214-1.470)	0.298	0.751(0.287-1.968)	0.582	0.698(0.297-1.643)	0.443
Pathological Tumor stage						
pT2	0.912(0.481-1.730)	0.782	0.943(0494-1.803)	0.862	1.013(0.547-1.881)	0.965
pT3	0.526(0.081-3.43)	0.559	1.052(0.190-5.834)	0.952	0.838(0.172-4.086)	0.832
Gleason score						
7	0.982(0.402-2.40)	0.969	0.902(0.353-2.305)	0.832	1.047(0.420-2.615)	0.914
8	1.052(0.314-3.533)	0.933	1.315(0.411-4.211)	0.629	1.164(0.380-3.568)	0.785
9	1.052(0.260-4.261)	0.942	1.263(0.287-5.566)	0.748	0.598(0.148-2.42)	0.517
10	2.105(0.108-40.874)	0.590	0		2.095(0.108-40.577)	0.592
Stage N1	0.842(0.173-4.111)	0.837	1.052(0.260-4.261)	0.942	0.897(0.239-3.379)	0.024

Cox proportional regression hazard model of the PBT group revealed that symptomatic presentation, greater tumor diameter, higher staging, high nuclear grade, presence of tumor necrosis, and lymph node involvement was a significant predictor of survival outcome (RFS, OS, and CSS) (*P*≤0.05), in nephrectomy patients (Table 6).

In cystectomy patients with PBT, symptomatic presentation, higher tumor staging, low-grade tumor, and lymph node involvement were seen as associated with increased all-cause mortality (RFS, OS, and CSS (*P*≤0.05)) (Table 7).

For prostatectomy patients who received PBT, blood transfusion was not a significant predictor for survival outcomes (Table 8).

## Discussion

Blood transfusion is a common intervention during surgeries. However, it may have long-term complications, especially in oncologic surgeries, which cannot be documented. After the introduction of transplant surgeries with the concept of immunosuppression clarified, blood transfusion with its various deleterious outcomes in patients undergoing radical curative surgeries for cancer has been demonstrated. Urological carcinomas rank high among them. The transfusion-produced energy can be expounded as one of the causes of the immunosuppressive activity of PBT (3). The transfused blood product contains abundant antigens, consequently leading to immune perturbations causing inflammatory and immunosuppressive systemic responses in patients (4). There is a discrepancy in the balance between cytokines and chemical mediators of inflammation in the transfused blood versus the reduced production of lymphocytes. This mechanism leads to the stimulation of cell-mediated cytokines, for example, interleukin. There is also a surge in the release of immunologically suppressive prostaglandins among patients undergoing surgeries with transfusion (5).

These studies inferred that at the cellular level, there is a decrease in the a) functional activity of natural killer (NK) cells. b) Cellular proliferation of T and B lymphocytes. c) Induction of T lymphocytes cells, and d) maturation along with the antigen-presenting capacity of dendritic cells. Most of the changes occurring at the cellular levels result from the infusion into the recipient's high concentrations of mediators of inflammation resulting in adverse responses in patients (2).

At the cellular level, there are numerous intra- and extra-cellular molecules which, by a complex biological metabolism, shoes an abnormal immune response occasionally after allogenic transfusion. (6.) 

"Fibrosarcoma in syngeneic mice and VX-2 neoplastic cell line in rabbits was diagnosed to be caused by white blood cells by blood transfusion in vitro. It was found that CD20+ and CD11+ dendritic cells were acting as stimulus uncontrolled growth in these inoculated experimental animals" (7).

 PBT promotes tumor proliferation by angiogenesis. It causes a significant increase in VEGF, and less production of endostatin resulting in vascular proliferation, experimentally proved in vitro (6).

Interestingly, various studies highlighted the fact that the timing of blood transfusion was associated with all-cause survival outcomes. Intraoperative blood transfusion had worse overall survival (OS) than postoperative blood transfusion. Since the patho-genesis is still not fully comprehended, it seems logically undebatable to find the probable reasons for this behavior (1,3,6).

The studies by Seon* et al. *(1), Linder* et al. *(8), Abu-Ghanem (9)* et al. *showed blood transfusion unfavorably affected cancer recurrence, cancer-specific death, overall survival in patients who had undergone nephrectomy for kidney cancer. Their studies demonstrate a highly significant prognostic relationship between blood transfusion and oncological survival outcomes in RCC cases. The studies by Jakobsen* et al. *(10), Moffat* et al. *(11), and Park* et al. *(6) fail to support the theory that blood transfusion increases the death rate after nephrectomy operations.

Our data confirm that in RCC patients, PBT negatively affects RFS, OS, and CSS in those with non-metastatic renal cancer undergoing therapeutic intervention. This is especially applicable for Renal Cell Carcinoma patients, especially those with clinical symptoms, older age, less hemoglobin value, higher creatinine value, and worse tumor grading. There should be clear-cut universal criteria for the limited use of PBT for better prognosis in such cases.

Cystectomy

Studies by Zamboni* et al. *(12), Chalfin* et al. *(13), Groeben* et al. *(14), radical cystectomy (RC) requires an intricate surgery with great dependency on blood transfusions. Several studies have elaborately discussed the result of blood transfusion in patients with urinary bladder carcinoma undergoing cystectomy procedures, focusing on outcomes. These studies have also investigated the acceptable timings of perioperative blood transfusion. Unfortunately, the literature fails to show a clear association between PBT and survival outcomes, with contrasting results in various studies. 

The studies by Volz* et al. *(15), Wang* et al. *(16), Moschi* et al. *(17), Cata* et al. *(18), Buchner* et al. *(19) showed the poor speculative impact of PBT on the prognostic outcome of patients with bladder carcinoma undergoing cystectomy. Our study found blood transfusion during radical cystectomy as an independent prognosis-affecting cause, specifically in those patients with a symptomatic presentation, higher tumor stage, and lymph node metastasis, causing increased all-cause mortality.

Prostate- Pushan* et al. *(20), Kim* et al. *(3), and Han* et al. *(21) reported that patients who received allogeneic RBCs perioperatively (predominantly in the postoperative period) did not have a cancer-related death or all-cause death in comparison with non-transfused patients. The studies by Boehm* et al. *(22), Yeoh* et al. *(23), Cata* et al. *(7) found that the surgeries with higher blood loss demanding more blood transfusions in relation to cancer recurrence to be irrelevant in prostate cancer patients in comparison to other malignant neoplasm undergoing surgical interventions. 

Our study failed to reveal any correlation between blood loss, blood transfusion, and survival outcomes in prostate cancer patients treated with radical prostatectomy (RP).

 Red blood cells undergo intracellular changes between blood collection and transfusion, predominantly after around the second to the third week of storage. Metabolites produced during this period depress immune functions (24). After an allogenic blood transfusion, the body's T cells signal B cell differentiation and maturation disorders resulting in less immunoglobulin secretion. The allogeneic RBCs and their degradation products also act as antigen-causing antigen-antibody reactions, consecutively leading to the loss of immunoglobulins (25).

 Our study highlights that perioperative blood transfusion is significantly associated with an increased risk of disease recurrence/cancer-specific mortality in nephrectomy and radical cystectomy patients. It also highlights the importance of the potential adverse outcomes associated with PBT. There is a need to lay down strict transfusion criteria and lessen the transfusion frequencies, particularly in oncological operations. Further studies will help explain the complex mechanism between transfusion and adverse reactions with special importance for each individualized treatment of vulnerable patients. 

## Conclusion

The indication for perioperative administration of blood products in oncourological surgeries should focus on 1) providing well-oxygenated blood 2) treating bleeding and coagulation disorders. A multidisciplinary team comprising physicians and surgeons should develop standard protocols monitoring the need for transfusions. These should be strictly implemented in oncological setups. The criteria for transfusion are personalized for each case, and a blanket rule cannot be applied everywhere, but the key is to minimize perioperative blood loss. The uses of recent technologies to replace blood loss with fluid administration and thoroughly assess the coagulation system guarantee better results in the long run 

 From our data, it is implied that PBT influences RFS, OS, and CSS in patients with renal and bladder cancer adversely undergoing curative operations. No significant correlation was identified in prostate cancer patients. Thus the systematic approach for limited use of PBT seems mandatory to improve postoperative survival. Prospective cohort studies in various tertiary institutions with longer postoperative follow-ups will yield better results. Clear, defined parameters for blood transfusions would guide the clinicians better. Autologous transfusion should be considered more frequently, where clinically possible, because of its immunological compatibility.

Limitations:

1) Patients who need blood transfusion are always clinically severe cases or have a general poor health condition, which may cause patient selection bias and influence the long-term outcomes. Because of practical ethical constraints, randomized trial has not yet been done in this area, as blood loss has no options other than transfusion 

2) Conclusion drawn from these studies should be interpreted cautiously as it may have been compromised by various confounding factors. The cohorts in our study were heterogeneous regarding tumor stage, frailty, and adjuvant therapy. We did not consider the effects of various other co-morbidities influencing blood transfusions. Postoperative infections could have been a dominant factor in cancer recurrence 3). The long duration of our study led to the development of many newer improved surgical treatments for urological tumors. Also, the transfusion requirements and component usage had changed considerably during the time frame causing certain discrepancies in the data. Future studies should address these questions.

## Conflict of Interest

The authors declared no conflict of interest.

## Funding

None.
